# Clinical and Neuroimaging Predictors of Posterior Circulation Stroke: A Retrospective Analysis of In-Hospital Features

**DOI:** 10.3390/brainsci16040418

**Published:** 2026-04-16

**Authors:** Rosalinda Calandrelli, Valerio Brunetti, Carlo Augusto Mallio, Eleonora Rollo, Daniele Vertulli, Luigi Ruscelli, Adriano Bonura, Francesca Santoro, Marco Sferruzzi, Sergio Soeren Rossi, Davide Norata, Francesco Motolese, Aldobrando Broccolini, Sabrina Anticoli, Fioravante Capone, Vincenzo Di Lazzaro, Fabio Pilato

**Affiliations:** 1 Radiology and Neuroradiology Unit, Department of Imaging, Radiation Therapy and Hematology, Fondazione Policlinico Universitario Agostino Gemelli IRCCS, Largo A. Gemelli, 1, 00168 Rome, Italy; 2 Unit of Neurology, Neurophysiology, Neurobiology and Psychiatry, Università Campus Bio-Medico of Rome, 00128 Rome, Italy; 3 Department of Neurosciences, Università Cattolica del Sacro Cuore, 00168 Rome, Italy; 4 UOC di Neurologia, Dipartimento di Neuroscienze, Organi di Senso e Torace, Fondazione Policlinico Universitario Agostino Gemelli IRCCS, 00168 Rome, Italy; 5 Research Unit of Radiology, Department of Medicine and Surgery, Università Campus Bio-Medico di Roma, 00128 Rome, Italy; 6 Fondazione Policlinico Universitario Campus Bio-Medico, 00128 Rome, Italy; 7 Center for Neurodegenerative Diseases and the Aging Brain, University of Bari Aldo Moro at Pia Fondazione “Card. G. Panico”, 73039 Tricase, Italy; 8 Stroke Unit, Department of Neuroscience, San Camillo Forlanini Hospital, 00152 Rome, Italy

**Keywords:** magnetic resonance imaging, stroke, large vessel occlusion, pc-ASPECT, modified Rankin Scale

## Abstract

**Highlights:**

**What are the main findings?**

- The prognosis of posterior circulation stroke is influenced not only by the infarct size but also by the specific artery involved, emphasizing the necessity of targeted neuroradiological assessment.
- Patients with large vessel occlusion (LVO) in posterior brain circulation, particularly those with basilar artery occlusion, showed more severe clinical presentation, more extensive bilateral lesions, and larger ischemic volumes compared to patients without LVO.

**What are the implications of the main findings?**

- The identification of specific clinical and imaging markers may enhance prognostic stratification and facilitate the development of personalized therapeutic strategies for patients with posterior circulation stroke.
- Predictors of poor outcome included ischemic lesion location and pre-stroke mRS, ischemic volume was associated only with short- term outcomes, whereas age and diabetes were associated with long-term outcomes.

**Abstract:**

**Objectives**: To investigate clinical and imaging predictors of short- and long-term outcomes in patients with posterior circulation stroke (PCS), with particular focus on infarct topography and ischemic burden. **Methods**: We conducted a retrospective multicenter observational study including 251 consecutive patients with acute PCS. All patients underwent CT angiography within 24 h and follow-up CT/MRI at 48–72 h. Clinical data, vascular risk factors, stroke severity (NIHSS), and functional outcome assessed by modified Rankin Scale (mRS), were collected. Short-term outcome was defined as mRS at discharge and long-term outcome as mRS at 3 months. Favorable outcome was defined as independence, graded as mRS 0–1. Imaging analysis included pc-ASPECTS, collateral scores, and quantitative ischemic volume assessment. Multivariable logistic regression was performed to identify independent predictors of outcome. **Results**: Among 251 patients, 105 (41.8%) had LVO. Patients with LVO presented with higher NIHSS scores, larger infarct volumes, and more frequent multiregional involvement. Basilar artery occlusion was associated with the most severe clinical and radiological profile. Infarct location, ischemic volume, baseline NIHSS, and pre-stroke mRS were independently associated with short-term outcome. For long-term outcome, age, infarct location, diabetes, and pre-stroke mRS remained significant predictors. LVO status and treatment variables were not independently associated with outcome. **Conclusions**: In PCS, outcome is primarily influenced by infarct topography and clinical factors rather than LVO status alone. Multiregional involvement and baseline disability are key determinants of prognosis. These findings underscore the need for PCS-specific prognostic models and highlight the importance of detailed imaging assessment beyond vessel occlusion.

## 1. Introduction

Ischemic stroke involving the posterior circulation (PCS) accounts for approximately 20% of all ischemic strokes (IS) [1] and differs from anterior circulation stroke in terms of clinical presentation and prognosis. These differences are largely attributable to the distinct cerebrovascular anatomy, lesion distribution, and the critical functions of brainstem structures [2,3].

The clinical severity and outcomes of PCS are highly variable and depend on a combination of patient-related factors, such as age and sex, and vascular characteristics, including anatomical variations in the vertebrobasilar system and the status of collateral circulation [2,4].

In anterior circulation stroke, baseline infarct extent and collateral status (CS) are well-established imaging predictors of clinical outcome in patients with large vessel occlusion (LVO) [5,6]. More recently, their prognostic value has also been explored in PCS, particularly in cases of basilar artery occlusion (BAO) [7,8,9].

Over the past years, research has predominantly focused on anterior circulation stroke (ACS), and patients with PCS were often excluded from major clinical trials targeting ACS [10,11]. However, growing interest in PCS has emerged more recently, in parallel with advances in acute stroke management [12].

In the emergency setting, CT-based imaging protocols are widely used due to their availability and rapid acquisition [13]. However, their sensitivity in detecting posterior fossa ischemia is limited by beam-hardening and bone-related artifacts, particularly in the inferior posterior fossa [14]. On the other hand, MRI in acute scenario is limited but it shows a higher sensitivity than CT for detecting posterior fossa ischemia [15].

To date, relatively few studies have specifically investigated PCS with a focus on imaging-defined topographic patterns, irrespective of the presence of arterial occlusion [16,17,18]. Moreover, only a limited number of studies have compared clinical, radiological features, and outcomes between PCS patients with and without LVO [19].

Given the distinct anatomical and physiological characteristics of the posterior circulation, we hypothesized that prognostic factors in PCS may differ from those established in anterior circulation stroke. Accordingly, the aims of this study were: (1) to evaluate differences in demographic characteristics, clinical risk factors, and neuroradiological findings between PCS patients with and without LVO; and (2) to identify clinical and imaging predictors of short- and long-term outcomes in PCS.

## 2. Methods

### 2.1. Study Design and Population

This was a retrospective, multicenter observational study conducted at two university hospitals. Institutional databases were systematically reviewed to identify and extract anonymized clinical and imaging data from eligible patients.

The study period spanned January 2021 to December 2023. The study protocol was approved by the local Ethics Committee (Prot. PAR 18.23) and conducted in accordance with the Declaration of Helsinki. Given the retrospective design and the use of fully anonymized data, the requirement for informed consent was waived in line with institutional regulations.

### 2.2. Inclusion and Exclusion Criteria

Patients were included if they met the following criteria:

- Diagnosis of acute posterior circulation stroke (PCS);
- Availability of CT angiography (CTA) within 24 h of symptom onset;
- Availability of follow-up CT and MRI performed within 48–72 h.

Exclusion criteria were:

- Transient ischemic attack (TIA);
- Anterior circulation stroke or mixed anterior/posterior stroke.

### 2.3. Data Collection

Baseline data included demographic characteristics (age, sex), vascular risk factors (hypertension, diabetes, atrial fibrillation, dyslipidemia, smoking, coronary artery disease, prior stroke/TIA), and stroke etiology classified according to TOAST criteria [20].

Stroke severity was assessed using the National Institutes of Health Stroke Scale (NIHSS) [21] at admission and discharge and Posterior Circulation Alberta Stroke Program Early CT Score (pc-ASPECTS) [7] were recorded by a vascular neurologist. Functional status was assessed using the modified Rankin Scale (mRS) [22].

Functional outcome was evaluated using the modified Rankin Scale (mRS):

- Pre-stroke (baseline);
- At discharge (short-term outcome);
- At 3 months (long-term outcome), assessed via structured telephone interview.

Favorable outcome was defined as independence graded as mRS 0–1. A sensitivity analysis using mRS 0–2 was also performed.

Patients received intravenous thrombolysis and/or mechanical thrombectomy according to the current guidelines [23].

### 2.4. Imaging Protocol

All patients underwent multimodal CT imaging, including non-enhanced CT and CTA, as described in the literature [24,25], using a 64 multi-slice CT. Baseline imaging consisted of CTA performed within 24 h of symptom onset. Follow-up imaging included CT and MRI performed within 48–72 h.

Acquisition parameters for CT were 120 kv and 300 mAs, and acquisition duration was 9 s. Acquisition parameters from CTA were 100 kV and 320 mAs, and acquisition duration was 21 s; after administration of 80 mL of contrast media at 4 mL/s flow, the acquisition was composed of three subsequent phases (separated by an interval of 8 s): the first phase (acquired in the arterial phase) extends from the aortic arch to the vertex, and the next two phases (acquired in the early and late venous phases) from the occipital foramen to the vertex.

All MRI studies were performed using a 1.5 T system equipped with a conventional quadrature head coil.

The imaging protocol included axial T1-weighted sequences (TR/TE = 500/15 ms; slice thickness = 4 mm) and T2-weighted sequences (TR/TE = 4000/100 ms; slice thickness = 4 mm), fluid-attenuated inversion recovery (FLAIR; TR/TE/TI = 11,000/140/2800 ms; slice thickness = 4 mm), diffusion-weighted imaging (DWI; TR/TE = 5400/87 ms; b value = 1000 s/mm^2^; slice thickness = 4 mm), susceptibility-weighted imaging (T2*-weighted echo-planar sequence; TR/TE = 50/12 ms; slice thickness = 3 mm), and three-dimensional time-of-flight MR angiography (TOF-MRA; TR/TE = 23/7 ms; slice thickness = 1.4 mm).

### 2.5. Radiological Assessment

**Figure 1:**
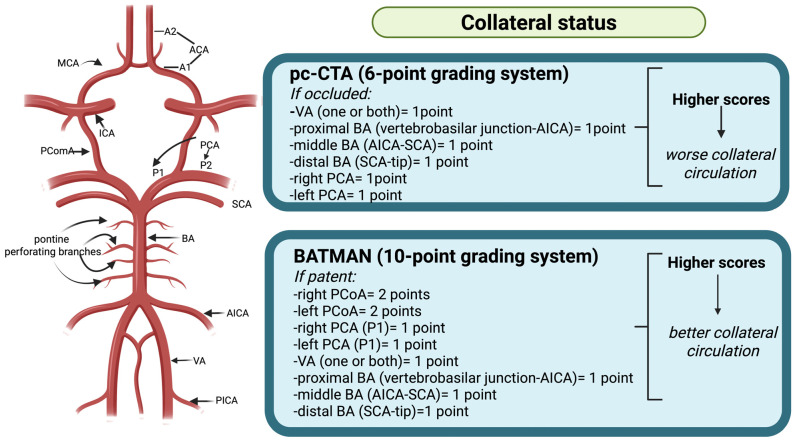
Evaluation of collateral status on computed tomography angiography (CTA) using the Posterior Circulation CTA (pc-CTA) score and basilar artery (BATMAN) score.

Posterior circulation ASPECTS (pc-ASPECTS) and collateral circulation scores (pc-CTA and BATMAN) [8,26,27] were independently assessed by two experienced neuroradiologists blinded to clinical data (Figure 1).

### 2.6. Ischemic Volume Analysis

Ischemic lesion volumes were segmented using ITK-SNAP software (version 4.4.0).

To account for anatomical variability across posterior circulation structures, lesion volumes were normalized using reference values derived from 20 age- and sex-matched healthy controls. While this approach enhances comparability across regions, its limitations are acknowledged.

### 2.7. Patient Classification

Patients were stratified according to:

- Presence or absence of LVO;
- Vessel involved (posterior cerebral artery, basilar artery, vertebral artery);
- Infarct distribution (supratentorial, cerebellar, brainstem, or multiregional).

### 2.8. Statistical Analysis

Categorical variables are presented as frequencies and percentages, while continuous variables are expressed as median and interquartile range (IQR).

Group comparisons were performed using the Mann–Whitney U test, Kruskal–Wallis test, or chi-square test, as appropriate.

Correlations were assessed using Pearson’s correlation coefficient. Multivariable logistic regression analyses were performed to identify independent predictors of outcome. Variables were selected based on clinical relevance and/or a *p*-value < 0.10 in univariable analyses. To ensure model stability, the number of events per variable (EPV) was considered, maintaining an EPV ≥ 10 whenever feasible.

Potential collinearity among predictors (including NIHSS, infarct volume, and pc-ASPECTS) was assessed using variance inflation factors (VIF), and variables showing significant collinearity were not included simultaneously in the same model.

Inter-rater reliability was evaluated using the intraclass correlation coefficient.

### 2.9. Missing Data and Bias Assessment

Three-month outcome data were available for 147 of 251 patients. To assess potential attrition bias, baseline characteristics were compared between patients with and without follow-up data. No significant differences were observed.

### 2.10. Power Considerations

Given the retrospective design, no “a priori” sample size calculation was performed. However, the relatively large cohort supports the robustness of the findings.

The data were analyzed with SPSS for Windows (version 25.0; IBM, Chicago, IL, USA); the level of significance was set at *p* < 0.05. To account for multiple comparisons, Bonferroni correction was applied where appropriate by adjusting the significance threshold according to the number of comparisons performed. The total number of comparisons considered for Bonferroni correction was 3, resulting in an adjusted significance threshold of *p* < 0.016.

## 3. Results

### 3.1. Patient Characteristics

A total of 251 patients were included, of whom 105 had LVO and 146 did not. Baseline characteristics are summarized in Table 1.

Patients with LVO were older, had shorter onset-to-imaging times, and more frequently presented with atrial fibrillation. They also exhibited higher NIHSS scores at both admission and discharge and were more likely to receive thrombolysis (Table 1 and Table 2).

### 3.2. Radiological Findings

Compared with non-LVO patients, those with LVO demonstrated significantly larger infarct volumes, more frequent multiregional involvement, higher rates of bilateral lesions, and increased incidence of hemorrhagic transformation. Interobserver agreement among radiologists was high (ICC = 0.81).

### 3.3. LVO Subgroup Analysis

Within the LVO group, BAO was associated with the most severe clinical and radiological profile, including higher NIHSS scores, larger brainstem infarcts, poorer collateral circulation, and a higher rate of thrombectomy.

**Figure 2:**
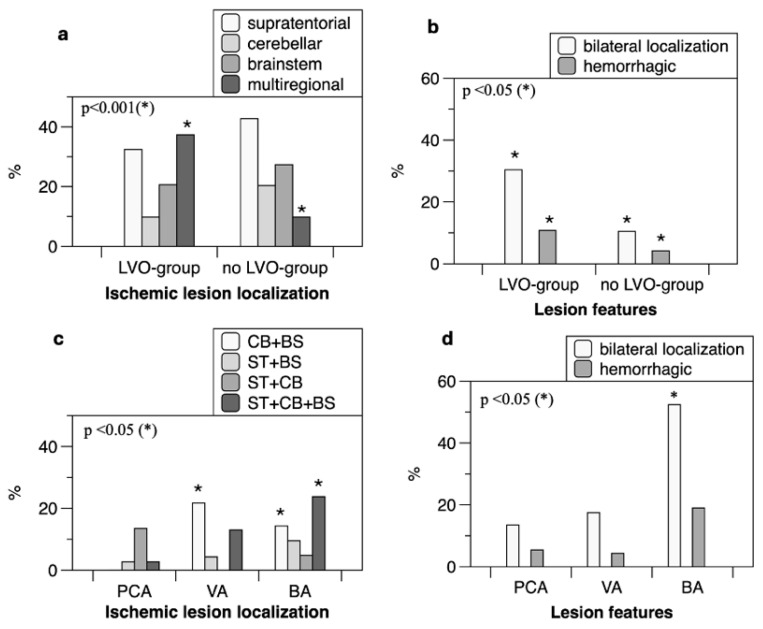
Stacked bar charts (**a**–**d**) display differences in MRI ischemic lesion localization and characteristics between patients with and without large vessel occlusion (LVO) in posterior circulation and among the three LVO patient subgroups. (**a**,**b**) Compared to the no-LVO group, the LVO group shows a higher rate of multiregional ischemic involvement, more frequent bilateral ischemic lesions, and a higher incidence of hemorrhagic infarction. * Indicates significance. (**c**,**d**) Among the three LVO subgroups (PCA, VA, and BA), patients with BA and VA occlusions more often exhibited combined brainstem and cerebellar ischemic lesions than those with PCA occlusion. Furthermore, patients with BA occlusion had a higher incidence of bilateral ischemic lesions than the other subgroups. * Indicates significance. MRI, Magnetic Resonance Imaging; PCA, posterior cerebral artery; VA, vertebral artery; BA, basilar artery.

**Figure 3:**
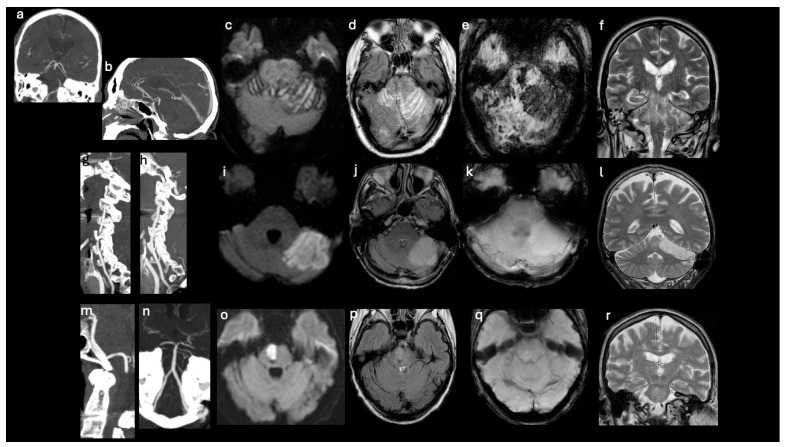
Topographic representation of ischemic lesions in patients with and without large vessel posterior circulation occlusion. CTA: (**a**,**b**,**g**,**h**,**m**,**n**); MRI: axial DWI (**c**,**i**,**o**); axial FLAIR (**d**,**j**,**p**); axial SWI (**e**,**k**,**q**); coronal T2 FSE (**f**,**l**,**r**). *Patient 1 (LVO)* (**a**–**f**). A 72-year-old man with a mid-basilar artery occlusion at CTA (**a**,**b**). Imaging shows an extensive, multiregional ischemic area involving the brainstem and cerebellar hemispheres characterized by T2 FSE, FLAIR, and DWI hyperintensity (consistent with cytotoxic edema) and SWI hypointensity (suggesting hemorrhagic infarction) (**c**–**f**). *Patients 2 (no-LVO) *(**g**–**l**). A 67-year-old man with a superior left cerebellar ischemic lesion, displaying T2 FSE, FLAIR, and DWI hyperintensity (indicative of cytotoxic edema) (**i**–**l**). No large vessel occlusion detected at CTA (**g**,**h**). *Patients 3 (no-LVO)* (**m**–**r**). A 66-year-old woman with an ischemic lesion on the right side of the pons, characterized by T2 FSE, FLAIR, and DWI hyperintensity (**o**–**r**). No large vessel occlusion detected at CTA (**m**,**n**). CTA, Computed Tomography Angiography; MRI, Magnetic Resonance Imaging; LVO, large vessel occlusion.

Posterior cerebral artery occlusion was primarily associated with supratentorial infarcts, while vertebral artery occlusion more frequently resulted in isolated brainstem involvement (Figure 2 and Figure 3).

### 3.4. Correlation Analysis

NIHSS scores showed a moderate negative correlation with pc-ASPECTS and a moderate positive correlation with brainstem infarct volume (r = 0.407).

These correlations, although statistically significant, were of moderate strength and should therefore be interpreted with caution.

### 3.5. Follow-Up Analysis

No significant differences in baseline characteristics were observed between patients with and without 3-month follow-up, suggesting limited attrition bias.

### 3.6. Treatment Analysis

Recanalization data (e.g., TICI scores) were not available. Therefore, treatment variables reflect whether treatment was performed rather than whether successful reperfusion was achieved (Table 3 and Table 4).

### 3.7. Predictors of Outcome

Multivariable analysis identified infarct location, ischemic volume, NIHSS, and pre-stroke mRS as independent predictors of short-term outcome.

For long-term outcome, age, infarct location, diabetes, and pre-stroke mRS emerged as independent predictors (Table 5).

**Figure 4:**
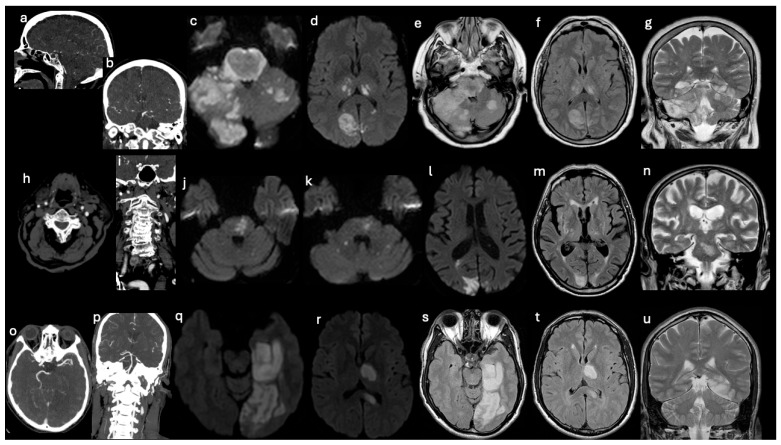
Topographic representation of ischemic lesions in three subgroups of patients with large vessel posterior circulation occlusion. CTA: (**a**,**b**,**h**,**i**,**o**,**p**); MRI: axial DWI (**c**,**d**,**j**,**k**,**l**,**q**,**r**); axial FLAIR (**e**,**f**,**m**,**s**,**t**); coronal T2 FSE (**g**,**n**,**u**). *Patient with basilar artery (BA) occlusion* (**a**–**g**). A 72-year-old man with mid-BA occlusion at CTA (**a**,**b**). Imaging shows a multiregional bilateral ischemic area involving the brainstem, cerebellar hemispheres and supratentorial regions including occipital lobes and thalami. The ischemic regions exhibit T2 FSE, FLAIR, and DWI hyperintensity (consistent with cytotoxic edema) and display parenchymal swelling (**c**–**g**). *Patient with vertebral artery (VA) occlusion* (**h**–**n**). A 75-year-old woman with left VA occlusion at CTA (**h**,**i**), shows multiregional ischemic involvement. Bilateral recent ischemic areas are present in the cerebellar hemispheres, brainstem, and occipital lobes (**j**–**n**). *Patient with posterior cerebral artery (PCA) occlusion* (**o**–**u**). A 79-year-old man with left PCA occlusion at CTA (**o**–**p**), displays an extensive supratentorial ischemic area involving the left temporo-occipital lobes, left thalamus, and the left portion of the corpus callosum (**q**–**u**). CTA, Computed Tomography Angiography; MRI, Magnetic Resonance Imaging.

LVO status and treatment variables were not independently associated with outcomes (Figure 2 and Figure 4).

### 3.8. Sensitivity Analysis

Sensitivity analysis using mRS 0–2 as the definition of favorable outcome yielded consistent results.

## 4. Discussion

This study provides a comprehensive evaluation of clinical and imaging predictors of outcome in posterior circulation stroke (PCS) within a multicenter cohort. The results underscore the marked heterogeneity of PCS and highlight that outcomes are determined by a complex interplay of clinical severity, infarct location, and patient-specific factors, rather than by LVO status alone.

Consistent with prior literature, patients with LVO presented with more severe neurological deficits, larger infarct volumes, and more extensive lesion distribution. In particular, BAO was associated with the most severe clinical and radiological profile, confirming its well-established role as the most critical subtype of PCS [28].

A key finding of this study is that infarct topography emerged as a stronger predictor of outcome than LVO status. Multiregional involvement, especially when affecting both brainstem and cerebellar structures, was independently associated with worse prognosis. This supports the notion that functional impairment in PCS is more closely related to the disruption of critical neuroanatomical networks than to the presence of vascular occlusion per se [29,30,31].

There is a knowledge gap in the literature between anterior and posterior stroke in terms of risk assessment, therapeutic options and outcome evaluation because patients with PCS have been underrepresented in research on acute stroke management because they were excluded from previous clinical trials [11,32,33], while only a few studies have investigated the clinical and radiological features of PCS and its prognosis [34,35,36].

Ischemic volume and baseline NIHSS were associated with short-term outcomes but did not retain significance in long-term outcome models. This discrepancy may reflect the unique anatomical and functional characteristics of posterior circulation structures, including a higher proportion of white matter and potential for functional adaptation [2,37]. However, given the observational nature of the study, these interpretations should be considered exploratory.

Age and diabetes were independently associated with long-term outcomes, in line with existing evidence emphasizing the impact of systemic comorbidities on recovery after stroke. These factors likely influence both acute vulnerability and long-term neuroplasticity.

Contrary to expectations, neither LVO status nor treatment variables were independently associated with outcome. This finding should be interpreted cautiously. Treatment allocation was not randomized, and patients undergoing thrombectomy typically had more severe strokes. Furthermore, the absence of recanalization data (e.g., TICI scores) precluded differentiation between successful and unsuccessful reperfusion, which is a critical determinant of outcome in stroke research [38].

Similarly to anterior circulation, the severity of neurological deficits at stroke onset, as measured by baseline NIHSS, correlated positively with brainstem ischemic volumes in the LVO group, indicating that more severe impairment was found in patients with larger brainstem ischemic areas [39,40].

The substantial loss to follow-up represents a potential limitation. However, the absence of significant baseline differences between patients with and without follow-up suggests that loss to follow-up was unlikely to introduce substantial selection bias.

The normalization of infarct volumes using a control cohort represents an effort to improve anatomical comparability across posterior circulation regions. Nonetheless, the relatively small number of controls and the lack of external validation limit the generalizability of this approach.

A formal a priori power calculation was not performed due to the retrospective design; however, the relatively large sample size strengthens the reliability of the observed associations. Importantly, due to the retrospective design, causal inferences cannot be drawn. All associations identified should therefore be interpreted with caution.

### Limitations

This study has several limitations, including its retrospective design, potential selection bias, lack of perfusion imaging, and absence of recanalization data. The relatively high rate of missing long-term follow-up data may also affect the robustness of outcome analyses. Additionally, the use of a strict definition of favorable outcome (mRS 0–1) may limit comparability with other studies, although sensitivity analyses confirmed the consistency of the findings.

## 5. Conclusions

Posterior circulation stroke is a highly heterogeneous condition in which infarct location, baseline clinical severity, and patient-related factors play a central role in determining outcome. Multiregional involvement and pre-stroke disability are key determinants of prognosis, whereas the impact of LVO appears less prominent than in anterior circulation stroke. These findings highlight the need for tailored prognostic models and therapeutic strategies specific to PCS.

## Figures and Tables

**Table 1:** Comparison of clinical and neuroradiological findings in posterior circulation ischemia with and without large vessel occlusion.

	LVO n. 105 (41.8%)	No-LVO n. 146 (58.2%)	*p*-Value
**Clinical data**			
Sex (M) n. (%)	65 (61.9%)	94 (64.4%)	0.688
Age (years) median [IQR]	75 [IQR 66–82]	71 [IQR 60–81]	**0.042**
Onset to CT-time (minutes) median [IQR]	258 [IQR 142–491]	418 [IQR 270–660]	**<0.001**
Hemorrhagic infarction n. (%)	11 (10.8%)	6 (4.2%)	**0.045**
Hypertension n. (%)	80 (76.2%)	96 (65.8%)	0.075
Atrial fibrillation n. (%)	37 (35.2%)	31 (21.2%)	**0.014**
Diabetes n. (%)	25 (23.8%)	39 (26.7%)	0.603
Dyslipidemia n. (%)	32 (30.5%)	62 (42.5%)	0.053
Chronic ischemic heart disease n. (%)	12 (11.4%)	21 (14.4%)	0.494
Carotid atherosclerosis n. (%)	22 (21.0%)	36 (24.7%)	0.492
COPD n. (%)	9 (8.6%)	5 (3.4%)	0.080
Previous stroke/TIA n. (%)	14 (13.3%)	17 (11.6%)	0.688
Antiplatelet at baseline n. (%)	27 (25.7%)	54 (37.0%)	0.060
Anticoagulants at baseline n. (%)	13 (12.4%)	14 (9.6%)	0.481
Statins n. (%)	21 (20.0%)	38 (26.0%)	0.267
NIHSS (baseline) median [IQR]	5 [IQR 3–12]	3 [IQR 1–5]	**<0.001**
NIHSS (discharge) median [IQR]	3 [IQR 1–8]	1 [IQR 0–3]	**<0.001**
Thrombolytic treatment median n. (%)	31 (29.5%)	19 (13%)	**0.001**
**Imaging data**			
Ischemic lesion on imaging n. (%)	102 (97.1%)	143 (97.9%)	**<0.001**
PC-ASPECT (1CT) median [IQR]	10 [IQR 9–10]	10 [IQR 9–10]	0.713
Total ischemic V. (1CT) median [IQR]	1.73 [IQR 0.26–3.74]	0.44 [IQR 0.11–1.46]	**0.008**
Supratentorial ischemic V. (1CT) median [IQR]	2.34 [IQR 0.58–3.81]	0.59 [IQR 0.26–2.32]	**0.026**
Cerebellar ischemic V. (1CT) median [IQR]	10.21 [IQR 1.97–29.48]	6.83 [IQR 2.04–13.30]	0.457
Brainstem ischemic V. (1CT) median [IQR]	4.81 [IQR 3.02–6.69]	2.07 [IQR 0.62–4.26]	0.081
PC-ASPECT (2CT) median [IQR]	8 [IQR 7–9]	9 [IQR 8–9]	**<0.001**
Total ischemic V. (MRI) median [IQR]	1.95 [IQR 0.44–4.53]	0.41 [IQR 0.14–1.43]	**<0.001**
Supratentorial ischemic V. (MRI) median [IQR]	2.83 [IQR 0.71–9.53]	0.56 [IQR 0.25–1.74]	**<0.001**
Cerebellar ischemic V. (MRI) median [IQR]	17.10 [IQR 4.00–31.29]	17.11 [IQR 2.50–25.43]	0.522
Brainstem ischemic V. (MRI) median [IQR]	7.44 [IQR 3.99–16.29]	4.96 [IQR 2.60–8.13]	**0.041**

M, male; n., number; (%), percentage; LVO, large vessel occlusion; CT, computed tomography; MRI, magnetic resonance imaging; PC-ASPECT, posterior circulation Alberta Stroke Program Early CT Score; V., normalized percentage Volume; TIA, transient ischemic attack; NIHSS, National Institutes of Health Stroke Scale; COPD, Chronic Obstructive Pulmonary Disease; IQR, Interquartile Range. (1CT): CTA performed within 24 h of symptom onset, (2CT) (MRI): Follow-up CT and MRI performed within 48–72 h. Bold value indicates *p* < 0.05.

**Table 2:** Comparison of clinical and neuroradiological multiple variables in posterior circulation ischemia with and without large vessel occlusion.

	LVO (n. 105)	No-LVO (n. 146)	*p*-Value
**Ischemic lesion on imaging n. (%)**	102 (97.1%)	143 (97.9%)	**<0.001**
Ischemic lesion localization			
-Supratentorial ischemia n. (%)	33 (32.4%)	61 (42.7%)	0.102
-Cerebellar ischemia n. (%)	10 (9.8%)	29 (20.3%)	**0.027**
-Brainstem ischemia n. (%)	21 (20.6%)	39 (27.3%)	0.230
-Cerebellar + Brainstem ischemia n. (%)	11 (10.8%)	5 (3.5%)	**0.023**
-Supratentorial + Brainstem ischemia n. (%)	6 (5.9%)	2 (1.4%)	0.052
-Supratentorial + Cerebellar ischemia n. (%)	7 (6.9%)	6 (4.2%)	0.359
-Supratentorial + Cerebellar + Brainstem ischemia n. (%)	14 (13.7%)	1 (0.7%)	**<0.001**
**Ischemic lesion side**			
-Right n. (%)	32 (31.4%)	67 (46.9%)	**0.015**
-Left n. (%)	39 (38.2%)	61 (42.7%)	0.488
-Bilateral n. (%)	31 (30.4%)	15 (10.5%)	**<0.001**
**Etiology**	105 (41.8%)	(146; 58.2%)	**<0.001**
-LAA n. (%)	38 (36.2%)	31 (21.2%)	**0.009**
-CE n. (%)	41(39%)	35 (24%)	**0.010**
-SAO or SVD n. (%)	0 (0%)	39 (26.7%)	**<0.001**
-SOC n. (%)	5 (4.8%)	8 (5.5%)	0.800
-SUC or unknown n. (%)	21 (20%)	33 (22.6%)	0.621

n. number; (%), percentage; LVO, large vessel occlusion; LAA, Large artery atherosclerosis; CE, Cardioembolism; SAO, Small artery occlusion; SVD, Small vessel disease; SOC, Stroke of other determined etiology; SUC, Stroke of undetermined etiology. Bold value indicates *p* < 0.05. This table includes only the significant radiological and clinical multiple variables from Table 1.

**Table 3:** Comparison of clinical and neuroradiological findings among LVO subgroups in posterior circulation ischemia.

	PCA-Occlusion n. 37 (35.2%)	BA-Occlusion n. 44 (41.9%)	VA-Occlusion n. 24 (22.9%)	*p* Value
**Clinical data**				
Sex (M) n. (%)	26 (70.3%)	25 (56.8%)	14 (58.3%)	0.425
Age (years) median [IQR]	76.5 [IQR 71–83]	73 [IQR 65–82]	75 [IQR 68–82]	0.597
Onset to CT-time (minutes)	253 [IQR 137–451]	236 [IQR 130–339]	476 [IQR 270–1260]	**0.015**
Etiology (TOAST criteria) n. (%)	37 (35.2%)	44 (41.9%)	24 (22.9%)	0.172
Hemorrhagic infarction n. (%)	2 (5.4%)	8 (19.0%)	1 (4.3%)	0.079
Hypertension n. (%)	28 (75.7%)	32 (72.7%)	20 (83.3%)	0.615
Atrial fibrillation n. (%)	11 (29.7%)	19 (43.2%)	7 (29.2%)	0.351
Diabetes n. (%)	9 (24.3%)	10 (22.7%)	6 (25.0%)	0.974
Dyslipidemia n. (%)	9 (24.3%)	11 (25.0%)	12 (50.0%)	0.061
Chronic ischemic heart disease n. (%)	3 (8.1%)	8 (18.2%)	1 (4.2%)	0.162
Carotid atherosclerosis n. (%)	12 (32.4%)	5 (11.4%)	5 (20.8%)	0.068
COPD n. (%)	3 (8.1%)	4 (9.1%)	2 (8.3%)	0.987
Previous stroke/TIA n. (%)	6 (16.2%)	6 (13.6%)	2 (8.3%)	0.674
Antiplatelet at baseline n. (%)	11 (29.7%)	10 (22.7%)	6 (25.0%)	0.769
Anticoagulants at baseline n. (%)	4 (10.8%)	8 (18.2%)	1 (4.2%)	0.230
Statins n. (%)	5 (13.5%)	12 (27.3%)	4 (16.7%)	0.273
NIHSS (baseline) median [IQR]	4 [IQR 3–8]	12.5 [IQR 5–42]	3 [IQR 2–4]	**<0.001**
NIHSS (discharge) median [IQR]	3 [IQR 1–8]	4 [IQR 1–20]	3 [IQR 1–3]	0.278
Thrombolytic treatment median n. (%)	12 (32.4%)	15 (34.1%)	4 (16.7%)	0.287
Mechanical thrombectomy n. (%)	5(13.5%)	32 (72.7%)	2 (8.3%)	**<0.001**
**Imaging data**				
Ischemic lesion on imaging n. (%)	37 (35.2%)	42 (40.0%)	23 (21.9%)	**<0.001**
PC-ASPECT (1CT) median [IQR]	9 [IQR 9–10]	10 [IQR 9–10]	10 [IQR 9–10]	0.137
Total ischemic V. (1CT) median [IQR]	2.94 [IQR 0.56–4.51]	0.48 [IQR 0.21–3.76]	0.91 [IQR 0.23–2.83]	0.117
Supratentorial ischemic V. (1CT) median [IQR]	3.36 [IQR 0.62–5.15]	1.03 [IQR 0.42–2.34]	2.26 [IQR 0.31–4.22]	0.427
Cerebellar ischemic V. (1CT) median [IQR]	11.58 [IQR 4.89–18.28]	17.71 [IQR 1.97–23.59]	6.43 [IQR 0.53–30.16]	0.958
Brainstem ischemic V. (1CT) median [IQR]	-	5.31 [IQR 3.16–26.08]	3.42 [IQR 0.70–5.71]	0.354
PC-ASPECT (2CT) median [IQR]	8 [IQR 8–9]	7.5 [IQR 6–8]	8 [IQR 7–9]	**0.014**
Total ischemic V. (MRI) median [IQR]	3.54 [IQR 0.77–9.11]	1.49 [IQR 0.55–3.51]	0.76 [IQR 0.23–1.95]	**0.010**
Supratentorial ischemic V. (MRI) median [IQR]	3.65 [IQR 1.17–9.53]	1.34 [IQR 0.37–10.37]	1.76 [IQR 0.77–4.24]	0.299
Cerebellar ischemic V. (MRI) median [IQR]	5.14 [IQR 1.18–17.17]	19.83 [IQR 4.00–34.84]	17.1 [IQR 6.30–32.71]	0.251
Brainstem ischemic V. (MRI) median [IQR]	4.66 [IQR 0.58–19.93]	11.71 [IQR 6.60–21.63]	4.34 [IQR 1.66–7.65]	**0.001**
Pc-CTA median [IQR]	1 [IQR 1–1]	2 [IQR 1–2]	1 [IQR 1–1]	**<0.001**
BATMAN median [IQR]	7 [IQR 7–7]	7 [IQR 6–8]	7 [IQR 7–8]	**0.008**

PCA, posterior cerebral artery; BA, basilar artery; VA, vertebral artery; M, male; n., number; (%), percentage; CT, computed tomography; PC-ASPECT, posterior circulation Alberta Stroke Program Early CT Score; V, normalized percentage Volume; TOAST, Trial of Org 10172 in Acute Stroke Treatment; NIHSS, National Institutes of Health Stroke Scale; COPD, Chronic Obstructive Pulmonary Disease; IQR, Interquartile Range; Pc-CTA, Posterior Circulation Computed Tomography Angiography; BATMAN, Basilar Artery Angiography Novel Score. Bold value indicates *p* < 0.05.

**Table 4:** Comparison of clinical and neuroradiological multiple variables among LVO subgroups in posterior circulation ischemia.

	PCA-Occlusion n. 37 (35.2%)	BA-Occlusion n. 44 (41.9%)	VA-Occlusion n. 24 (22.9%)	PCA vs. BA-Occlusion (*p* Value)	PCA vs. VA-Occlusion (*p* Value)	BA vs. VA-Occlusion (*p* Value)
Onset to CT-time (minutes)	253 [IQR 137–451]	236 [IQR 130–339]	476 [IQR 270–1260]	0.317	0.052	**0.004**
PC-ASPECT (MRI) median [IQR]	8 [IQR 8–9]	7.5 [IQR 6–8]	8 [IQR 7–9]	**0.005**	0.698	0.065
Total ischemic V. (MRI) median [IQR]	3.54 [IQR 0.77–9.11]	1.49 [IQR 0.55–3.51]	0.76 [IQR 0.23–1.95]	0.178	**0.003**	0.041
Brainstem ischemic V. (MRI) median [IQR]	4.66 [IQR 0.58–19.93]	11.71 [IQR 6.60–21.63]	4.34 [IQR 1.66–7.65]	0.258	0.966	**<0.001**
**Ischemic lesion localization on imaging** n. (%)	37 (35.2%)	42 (40.0%)	23 (21.9%)			
-Supratentorial ischemia n. (%)	28 (75.7%)	5 (11.9%)	0 (0%)	**<0.001**	**<0.001**	0.085
-Cerebellar ischemia n. (%)	1 (2.7%)	5 (11.9%)	4 (17.4%)	0.123	0.045	0.540
-Brainstem ischemia n. (%)	1 (2.7%)	10 (23.8%)	10 (43.5%)	**0.007**	**<0.001**	0.100
-Cerebellar+ Brainstem ischemia n. (%)	0 (0%)	6 (14.3%)	5 (21.7%)	**0.017**	**0.003**	0.443
-Supratentorial+ Brainstem ischemia n. (%)	1 (2.7%)	4 (9.5%)	1 (4.3%)	0.214	0.730	0.454
-Supratentorial+ Cerebellar ischemia n. (%)	5 (13.5%)	2 (4.8%)	0 (0%)	0.172	0.066	0.288
-Supratentorial+ Cerebellar+ Brainstem ischemia n. (%)	1 (2.7%)	10 (23.8%)	3 (13%)	**0.007**	0.118	0.299
**Ischemic lesion side**						
-Right n. (%)	10 (27%)	11 (26.2%)	11 (47.8%)	0.933	0.101	0.078
-Left n. (%)	22 (59.5%)	9 (21.4%)	8 (34.8%)	**<0.001**	0.063	0.241
-Bilateral n. (%)	5 (13.5%)	22 (52.4%)	4 (17.4%)	**<0.001**	0.683	**0.006**
**Pc-CTA** median [IQR]	1 [IQR 1–1]	2 [IQR 1–2]	1 [IQR 1–1]	**<0.001**	0.634	**<0.001**
**BATMAN** median [IQR]	7 [IQR 7–7]	7 [IQR 6–8]	7 [IQR 7–8]	**0.008**	0.798	**0.016**
**NIHSS** (baseline) median [IQR]	4 [IQR 3–8]	12.5 [IQR 5–42]	3 [IQR 2–4]	**<0.001**	0.042	**<0.001**

PCA, posterior cerebral artery; BA, basilar artery; VA, vertebral artery; n., number; (%), percentage; CT, computed tomography; PC-ASPECT, posterior circulation Alberta Stroke Program Early CT Score; V, normalized percentage Volume; NIHSS, National Institutes of Health Stroke Scale; IQR, Interquartile Range; Pc-CTA, Posterior Circulation Computed Tomography Angiography; BATMAN, Basilar Artery Angiography Novel Score. Bold value indicates *p* < 0.05. This table considers only significant radiological and clinical findings showing multiple variables in Table 3. *p* value < 0.017 is significant according to the Bonferroni correction.

**Table 5:** Binary logistic regression analysis of clinical outcome at discharge and at 3 months of follow-up in patients with posterior circulation ischemia.

Clinical Outcome at Discharge				
	Confidence Interval 95%			
	OR	Lower	Upper	*p*-Value
Age (years)	0.978	0.953	1.005	0.107
Sex (M/F)	0.975	0.494	1.924	0.941
LVO (yes/no)	1.247	0.406	3.832	0.700
Pc-CTA	1.078	0.545	2.135	0.828
Ischemic lesion localization	0.768	0.620	0.952	**0.016**
Total ischemic V. (MRI)	0.893	0.803	0.992	**0.035**
AF	1.309	0.590	2.906	0.507
Diabetes	0.745	0.359	1.546	0.429
Acute Treatment (Thrombolytic/thrombectomy)	1.064	0.451	2.510	0.888
NIHSS baseline	0.873	0.800	0.951	**0.002**
Pre-mRS	0.292	0.160	0.535	**<0.001**
**Clinical Outcome at 3 Months of Follow-Up**				
	**Confidence Interval 95%**			
	**OR**	**Lower**	**Upper**	***p*** **-Value**
Age (years)	0.937	0.899	0.977	**0.002**
Sex (M/F)	1.162	0.421	3.206	0.772
LVO (yes/no)	1.368	0.226	8.277	0.733
Pc-CTA	1.141	0.474	2.743	0.768
Ischemic lesion localization	0.589	0.431	0.806	**<0.001**
Total ischemic V. (MRI)	1.007	0.874	1.159	0.928
AF	0.407	0.134	1.230	0.111
Diabetes	0.314	0.104	0.947	**0.040**
Treatments (Thrombolytic/thrombectomy)	2.367	0.521	10.757	0.265
NIHSS baseline	0.934	0.864	1.010	0.089
Pre-mRS	0.339	0.178	0.647	**0.001**

LVO, large vessel occlusion; OR, odds ratio; Pc-CTA, posterior circulation computed tomography angiography score; M, male; F, female; V., normalized percentage Volume; AF, atrial fibrillation; NIHSS, National Institutes of Health Stroke Scale; Pre-mRS, modified Rankin Scale score at the time of clinical onset. Ischemic lesion localization: isolated vs. multiregional ischemia. The clinical outcome at discharge and at 3 months of follow-up (mRS-discharge, mRS-3 months) was dichotomized into excellent (mRS 0–1) and non-excellent (mRS 2–6). About mRS-discharge, data were available for all patients with posterior circulation ischemia (n. 251 pts) while about mRS-3 months data were available for 147 out of 251 patients with posterior circulation ischemia. Bold value indicates *p* < 0.05.

## Data Availability

The original contributions presented in this study are included in the article. Further inquiries can be directed to the corresponding author.
